# The Combination of Gastroschisis, Jejunal Atresia, and Colonic Atresia in a Newborn

**DOI:** 10.1155/2015/129098

**Published:** 2015-06-09

**Authors:** Zachary Bauman, Victor Nanagas

**Affiliations:** ^1^Henry Ford Macomb Hospital, Clinton Township, MI 48038, USA; ^2^Dayton Children's Hospital, Dayton, OH 45404, USA

## Abstract

We encountered a rare case of gastroschisis associated with jejunal atresia and colonic atresia. In our case, the jejunal atresia was not discovered for 27 days after the initial abdominal wall closure. The colonic atresia was not discovered for 48 days after initial repair of the gastroschisis secondary to the rarity of the disorder. Both types of atresia were repaired with primary hand-sewn anastomoses. Other than the prolonged parenteral nutrition and hyperbilirubinemia, our patient did very well throughout his hospital course. Based on our case presentation, small bowel atresia and colonic atresia must be considered in patients who undergo abdominal wall closure for gastroschisis with prolonged symptoms suggestive of bowel obstruction. Our case report also demonstrates primary enteric anastomosis as a safe, well-tolerated surgical option for patients with types of intestinal atresia.

## 1. Introduction

Atresia of the colon is an uncommon cause of intestinal obstruction in the newborn patient, with classic etiologic factors resulting from intrauterine mesenteric vascular obstruction associated with internal hernia, volvulus, intussusception, or strangulation in tight gastroschisis [1]. It occurs between 1.8 and 15 percent of all patients affected by atresia of the bowel, with most studies quoting less than 10 percent of patients reviewed [2]. Reported incidences range from 1 in 1500 to 1 in 66,000 live births [3, 4]. Furthermore, approximately 5 to 15 percent of all infants born with gastroschisis will have intestinal stenosis or atresia resulting in increased time to full enteral feeds, prolonged parenteral nutrition, increased hospital stay, and increased mortality [5, 6]. Isolated colonic atresia is an extremely rare condition with limited information published about management and outcomes. Here, we report an even greater rarity in which a patient born with a known gastroschisis was later found to have both jejunal atresia and colonic atresia.

## 2. Case Report

A 35-week-old gestational age male weighing 2,495 grams was admitted to our neonatal intensive care unit (NICU) shortly after he was born secondary to previously diagnosed gastroschisis. The child was born to a 17-year-old gravida 1 para 1 female. The pregnancy was uncomplicated other than a traumatic event that occurred at 17 weeks resulting in placental abruption but no loss of the pregnancy. The mother did receive appropriate prenatal care. The child was born via an uncomplicated vaginal delivery with APGAR scores of 9 and 9 at one and five minutes, respectively. There is no family history of gastroschisis or intestinal atresia. The mother's only medical history included celiac disease.

Upon presentation to the NICU, the child was examined by the surgical service and deemed to be an appropriate candidate for reduction of the gastroschisis and primary closure of the abdominal wall defect. Only a small amount of bowel was protruding from the abdominal wall defect without signs of compromised bowel viability. The patient was subsequently taken to the operating room where a nasogastric tube was placed for decompression. Furthermore, the rectum of the patient was emptied of meconium by gentle anal dilatation and irrigation, providing standard of care prior to reduction and closure of the abdomen for the gastroschisis. The patient's gastroschisis was reduced and the abdominal wall was primarily closed without any complications. The extracorporeal bowel was found to be mildly edematous with minimal inflammatory peel and no signs of atresia. The patient was transferred back to the NICU where he was extubated within the first 24 hours postoperatively. A peripherally inserted central catheter was placed on postoperative day one and the patient was started on total peripheral nutrition (TPN), awaiting return of bowel function before starting enteral feeds. Postoperatively, the patient never developed any signs or symptoms of abdominal compartment syndrome or necrotizing enterocolitis. The patient was continued on IV antibiotics for a total of 72 hours from the date of surgery.

**Figure 1:**
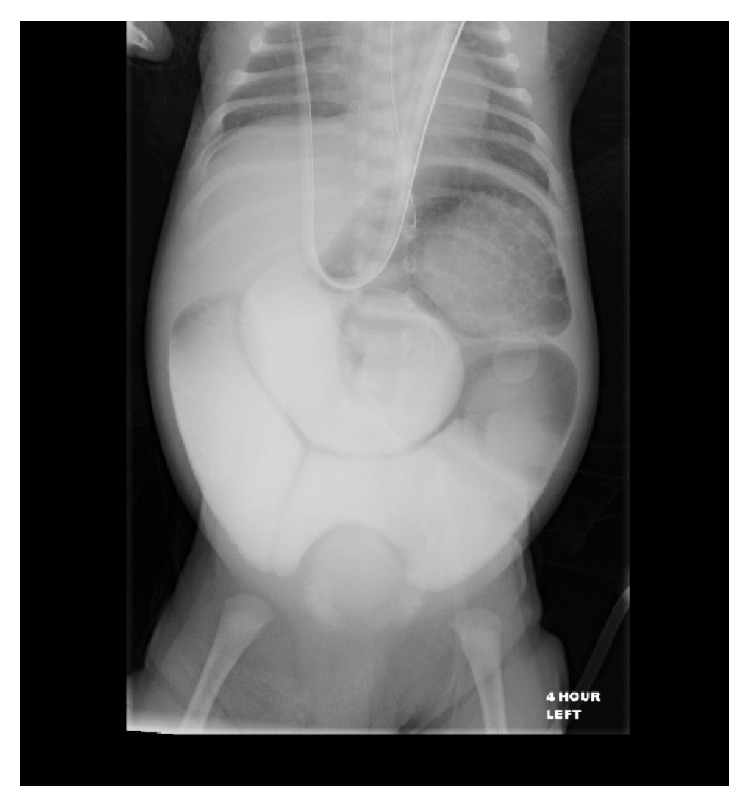
Upper gastrointestinal small bowel follow-through at 4 hours showing dilated loops of proximal small bowel and no progression of contrast through to the colon, suggestive of small bowel obstruction.

The patient remained hemodynamically stable with nasogastric decompression and parenteral nutrition. He continued to demonstrate high output from the nasogastric tube, which, over the course of his first three weeks of life, became more mucus-like and nonbilious. His abdomen remained mildly distended but, as Snyder et al. pointed out, patients can often suffer from severe ileus for up to 3 to 4 weeks after closure of the gastroschisis [7]. Further studies have also demonstrated prolonged ileus from closure of a gastroschisis. A study from 2000 by Driver et al. showed the median time to full oral feedings and resolution of ileus was 30 days (range: 5 to 160 days) [8] and a 2011 study by Bradnock et al. showed a median duration of 21 days (range: 9 to 39 days) to reach full oral intake [9]. For this reason, we never became alarmed as we felt our patient was just suffering a prolonged ileus. However, as the patient approached four weeks since the initial gastroschisis closure, the concern for a possible bowel obstruction became evident as the patient never developed any further bowel function since the original passage of meconium at the initial surgery. Therefore, an upper gastrointestinal study with small bowel follow-through was obtained (Figure 1). The study demonstrated multiple dilated loops of proximal small bowel, consistent with a small bowel obstruction. Therefore, on day of life 27, the decision was made to take the patient back to the operating room for exploratory laparotomy.

After an extensive enterolysis, it was found that the patient had type III proximal jejunal atresia, which is when the blind ends of bowel are separated by a V-shaped defect of the mesentery [1]. Eight centimeters of the distal atretic jejunum was resected secondary to questionable viability; however, the remaining bowel appeared healthy and without inflammatory peel. A catheter was inserted into the distal atretic enterotomy and sterile normal saline was injected, demonstrating patency of the bowel through to the ascending colon. At this point, a primary end-to-end hand-sewn enteroenterostomy was created followed by a proximal jejunal plication secondary to the size difference of the two ends of bowel. The abdomen was primarily closed and the patient was transferred to the NICU in a stable condition.

**Figure 2:**
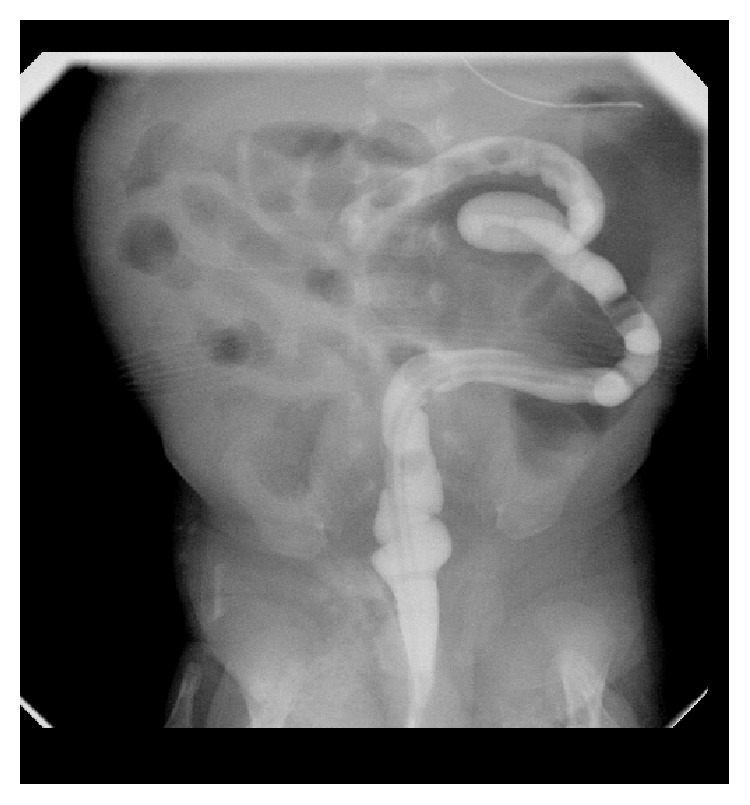
Barium enema showing microcolon and no progression of contrast proximal to the midtransverse colon suggestive of colonic obstruction.

**Figure 3:**
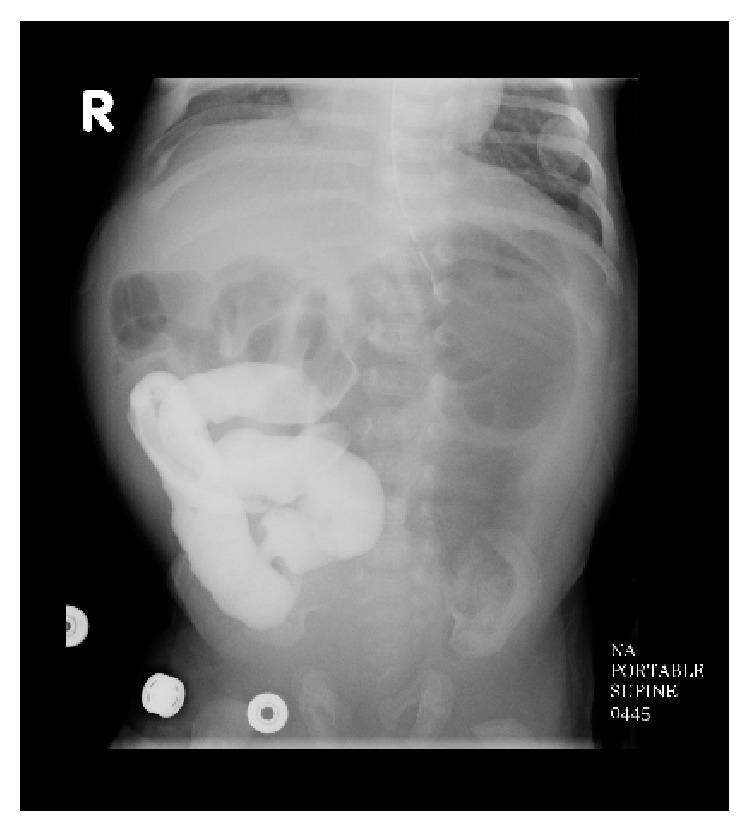
Upper gastrointestinal small bowel follow-through showing no progression of contrast beyond the right side of the abdomen after 4 days suggestive of obstruction. Previous enteroenterostomy appears patent.

Once again, the patient did well postoperatively. His TPN was continued and the nasogastric tube remained in place for decompression secondary to the anticipated postoperative ileus. Three weeks following the second surgery, there was again concern for an obstructive process, as the patient was still demonstrating high, nonbilious output from the nasogastric tube with chronic abdominal distension and no progression of bowel function. Furthermore, the patient also developed elevated bilirubin levels due to the extended period of time receiving TPN. At this point, we attributed the prolonged ileus to the previous two extensive surgeries experienced by the patient; however, in retrospect, we probably observed the patient too long this second time. The average postoperative ileus for jejunal atresia is approximately 5 days with a range of 3 to 10 days [10]. Additional imaging, such as an abdominal ultrasound, may have been beneficial during this time to help determine why the patient was not having bowel function. Nonetheless, a barium enema was obtained (Figure 2) after our allotted observation time demonstrating no progression of contrast beyond the midtransverse colon as well as significant microcolon, highly suggestive of colonic obstruction. Furthermore, an upper gastrointestinal study with small bowel follow-through was again performed (Figure 3). It demonstrated patency of the previously created jejunojejunostomy and propagation of contrast into the distal small bowel and proximal colon but was also suggestive of a distal colonic obstruction. Due to a work-up and clinical presentation highly suggestive of a colonic obstruction, the patient was taken back to the operating room on day of life 48 for a second exploratory laparotomy.

Again, extensive enterolysis was performed after entrance into the abdominal cavity. The previous jejunojejunostomy with the proximal plication was found to be patent. The bowel was followed to the colon where we discovered type II transverse colonic atresia, which is when the blind ends of bowel are separated by a fibrous cord [1]. An enterotomy was made in the distal atretic segment and 60cc of normal saline was injected into the distal colon with conformation of patency demonstrated by fluid excreted by the anus. The decision was made to perform a hand-sewn end-to-end anastomosis by spatulating the distal atretic segment. Once this was completed, the abdomen was closed and the patient was transferred to the NICU in a stable condition. The patient tolerated the procedure well.

The patient was continued on TPN and his nasogastric tube remained in place. A few days after the final surgery, the patient finally had his first bowel movement. Enteric tubes feeds were introduced slowly as his TPN was weaned. With the introduction of enteric feeds and weaning of TPN, the patient's hyperbilirubinemia gradually resolved.

## 3. Discussion

Although the incidence of gastroschisis, jejunal atresia, and colonic atresia found concomitantly in the same patient is not well defined in the current literature, only a signal case report has been described where one of a pair of dichorionic, diamniotic twins was found to have all three anomalies [6]. Diagnosis of intestinal atresia at the initial presentation of gastroschisis can be difficult secondary to a thick inflammatory peel covering the bowel [7], as well as poor visualization of bowel within the peritoneal cavity. Similar to the review by Kronfli et al., our missed intestinal atresia patient continued to demonstrate abdominal distension with prolonged feeding intolerance and high nasogastric tube output [5]. We were, however, able to recognize this obstructive jejunal atresia within 27 days of life, as opposed to the 41–57-day range cited in this review [5].

Our management of this patient was certainly not ideal as the patient required three operations in total. We believe this to be related to the rarity of having all three anomalies simultaneously. The classic surgical approach to managing patients with both gastroschisis and small bowel atresia has been to create a stoma in the acute setting with delayed primary anastomosis after a period of nasogastric decompression [5]. A similar surgical approach has been described for colonic atresia in which a primary anastomosis is performed for colonic atresia found proximal to the splenic flexure, and a colostomy with delayed anastomosis is performed for colonic atresia distal to the splenic flexure [1]. More recently, bowel resection with primary anastomosis has been demonstrated as a reasonable treatment for patients with gastroschisis and intestinal atresia [1, 5]. Of course, this is only possible when the bowel is healthy enough to undergo primary anastomosis, which is left to the judgment of the pediatric surgeon [7].

In our case presentation, the types of intestinal atresia were not initially diagnosed at abdominal wall closure secondary to a lack of bowel protruding through the abdominal wall defect and a low suspicion. Unfortunately, during our patient's second operation, transverse colon atresia was missed resulting in a third operation at day of life 48. Etensel et al. demonstrated in their meta-analysis of 224 cases of colonic atresia a statistically significant increase in mortality if time from birth to colonic atresia repair is greater than 72 hours [1]. Although our case does not support this increase in mortality from delayed repair, our patient was not without multiple comorbidities including hyperbilirubinemia, chronic TPN requirements, extended period of nasogastric tube insertion with high volume output, constant electrolyte abnormalities, inappropriate weight gain, and intestinal motility dysfunction.

Isolated colonic atresia is very uncommon, meaning its association with gastroschisis and jejunal atresia is even more so. Although the management of gastroschisis and intestinal atresia still remains contentious among various pediatric surgeons, the ultimate goal of restoring normal bowel function remains the focus of care for these patients. Healthcare providers must have a high suspicion of intestinal atresia associated with gastroschisis, something we lacked throughout this case, if neonates demonstrate intractable bilious vomiting, abdominal distension, and feeding intolerance on physical examination. We delayed the second operation because what we felt was a prolonged ileus from the gastroschisis itself, but that was obviously an incorrect assumption. Once the jejunal atresia was discovered, diagnostic dilemmas such as the false positive intraoperative saline injection test, not completely imaging the rectum and colon prior to surgery, and the initial passage of meconium during the initial gastroschisis closure plagued us causing the patient to require a third operation. Additionally, once the jejunal atresia was discovered, we should have had a higher suspicion for colonic atresia as jejunal atresia is associated with a greater number of simultaneous congenital malformations than ileal atresia [10].

The diagnosis of gastroschisis is fairly obvious and often diagnosed prior to birth. Intestinal atresia, however, can be more challenging, especially in the presence of other pathologic abnormalities. Although the use of both upper and lower contrast gastrointestinal studies should be included in the work-up of intestinal atresia, simply monitoring of bowel movements is extremely sensitive in assessing bowel obstruction. As clinicians, we were misled by the passage of a small amount of meconium at the initial surgery followed by the already known extended ileus that can result from these various pathologies. Utilizing bowel movement frequency with appropriate imaging modalities will allow for early and more accurate diagnosis of obstructive pathology in neonates, which would have been very beneficial for the patient described in our case report. Once intestinal atresia is diagnosed, time should not be wasted in repairing the obstructive pathology in order to decrease morbidity and mortality. When patients with such anomalies are managed appropriately, outcomes are generally favorable.
